# e-CBT (myCompass), Antidepressant Medication, and Face-to-Face Psychological Treatment for Depression in Australia: A Cost-Effectiveness Comparison

**DOI:** 10.2196/jmir.4207

**Published:** 2015-11-11

**Authors:** Daniela Solomon, Judith Proudfoot, Janine Clarke, Helen Christensen

**Affiliations:** ^1^ Black Dog Institute University of New South Wales Sydney Australia

**Keywords:** cost-utility analysis, depression, self-help, computer-assisted therapy

## Abstract

**Background:**

The economic cost of depression is becoming an ever more important determinant for health policy and decision makers. Internet-based interventions with and without therapist support have been found to be effective options for the treatment of mild to moderate depression. With increasing demands on health resources and shortages of mental health care professionals, the integration of cost-effective treatment options such as Internet-based programs into primary health care could increase efficiency in terms of resource use and costs.

**Objective:**

Our aim was to evaluate the cost-effectiveness of an Internet-based intervention (myCompass) for the treatment of mild-to-moderate depression compared to treatment as usual and cognitive behavior therapy in a stepped care model.

**Methods:**

A decision model was constructed using a cost utility framework to show both costs and health outcomes. In accordance with current treatment guidelines, a stepped care model included myCompass as the first low-intervention step in care for a proportion of the model cohort, with participants beginning from a low-intensity intervention to increasing levels of treatment. Model parameters were based on data from the recent randomized controlled trial of myCompass, which showed that the intervention reduced symptoms of depression, anxiety, and stress and improved work and social functioning for people with symptoms in the mild-to-moderate range.

**Results:**

The average net monetary benefit (NMB) was calculated, identifying myCompass as the strategy with the highest net benefit. The mean incremental NMB per individual for the myCompass group was AUD 1165.88 compared to treatment as usual and AUD 522.58 for the cognitive behavioral therapy model.

**Conclusions:**

Internet-based interventions can provide cost-effective access to treatment when provided as part of a stepped care model. Widespread dissemination of Internet-based programs can potentially reduce demands on primary and tertiary services and reduce unmet need.

## Introduction

Depressive disorders are highly prevalent [1], with an average lifetime prevalence of 14.6% [2]. The effects of these disorders extend beyond mental health to include diminished quality of life and functioning and increased mortality and medical morbidity for individuals [3], and substantial economic loss for society [4,5]. Antidepressant medication and cognitive behavior therapy (CBT) are established treatments for depressive disorders; however, current effective coverage is low and the economic burden attributable to preventable depressive disorders is substantial [6,7]. This situation necessitates alternative cost-effective models of care for people with depressive disorders.

Internet-delivered psychological interventions can facilitate broad access to evidence-based treatments and are popular with users and clinically effective, with outcomes equivalent to face-to-face therapies [8-10]. With large effect sizes and reduced human resource demands (ie, no or minimal therapist input), these interventions are also likely to substantially reduce treatment costs for individuals and society [11,12]. Nevertheless, research into the economic consequences of Internet-delivered interventions is in its infancy. While evidence supports the low marginal costs of providing therapy via the Internet [13-16], the cost-effectiveness of treatment models incorporating Internet-delivered psychotherapies remains largely unexplored. This lack of evidence provides a major impediment to the integration of Internet-delivered interventions into mainstream models of health service provision for depressive disorders.

Current international guidelines recommend a “stepped-care” approach to treatment of depressive disorders. In a stepped-care model, low-intensity interventions are offered to people initiating treatment for persistent subthreshold depressive symptoms or mild-to-moderate depression [17,18], and antidepressant medication and/or face-to-face psychological therapy are offered to those with moderate or severe depression or with ongoing symptoms following an initial low-intensity program. Low-intensity programs include CBT-based guided and unguided self-help, for example CBT delivered via the Internet.

In a recently conducted large-scale randomized controlled trial (RCT), Proudfoot et al [8] showed that a fully automated CBT-based public health intervention combining mobile phone and Web technology, myCompass, effectively reduced symptoms of depression, anxiety, and stress and improved work and social functioning for people with symptoms in the mild-to-moderate range compared with waitlist and placebo controlled conditions. Details of the myCompass trial have been published elsewhere [8]. In this study, we used data from the trial to examine the cost-effectiveness of myCompass when incorporated into a stepped-care approach to depression treatment. Specifically, we compared the cost-effectiveness of myCompass to treatment as usual (TAU; in Australia this is antidepressant medication) and face-to-face CBT, with a view to understanding the cost implications of incorporating this type of intervention into a stepped-care plan for community-based depression management. To our knowledge, this is the first direct comparison of this type undertaken using Australian health system costs and structure.

## Methods

### The Clinical Model

The chosen stepped-care model reflects international and Australian guidelines. International guidelines refer to specific criteria to define treatment response and symptom remission [19]; “response” is generally defined as >50% reduction in symptoms from commencement of treatment per standardized rating scale [20]. “Remission” is generally defined as an asymptomatic state [20]. However, major international guidelines are vague on the specific methods and measures used to track treatment progress [18,21-23]. In the myCompass trial, symptoms were measured using the Depression Anxiety Stress Scale (DASS) [24]. In the trial, an outcome of ˂27 on the DASS scale reflected a normal (asymptomatic) profile and is assumed here as equivalent to remission on the Hamilton Rating Scale for Depression (0-7) [20]. Probabilities for the “remission” and “maintenance” states in the model were derived from the proportion of myCompass trial participants with scores on the DASS in the normal range at the post-treatment and follow-up assessments.

As described previously, for stepped-care models all patients with mild-to-moderate symptoms start with the same low-intensity intervention. Symptom progress is monitored, and only those with inadequate improvement are offered a higher-intensity intervention. International guidelines recommend that when mild or moderate depression is non-responsive to low-intensity treatments, then antidepressant or face-to-face psychological therapy (eg, CBT or interpersonal psychotherapy) should be offered [17,18,25]. However, due to service delivery barriers, including insufficient psychological services and low workforce numbers, patients are commonly offered antidepressants in the initial stages of treatment [26,27].

In accordance with these guidelines, we examined a stepped-care model that included myCompass as a first step. While current Australian guidelines do not make explicit reference to Internet-delivered therapies, the self-guided nature of myCompass means that it fits very well with current definitions of low-intensity interventions (ie, self-help therapy). In the Australian health system, most people seeking treatment for depression are referred to services by a general practitioner, so this is also reflected in our model.

### Decision-Analytic Model

**Figure 1:**
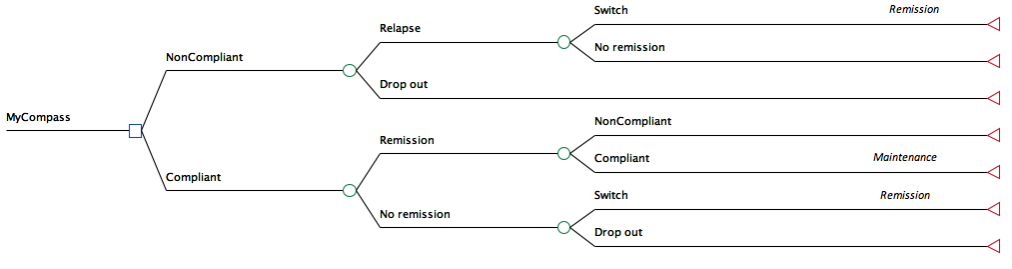
Partial decision tree structure (subtree is repeated for each arm of the model).

A decision tree model (Figure 1) for the treatment of mild-to-moderate depression was constructed using a cost utility framework to show both costs and health outcomes over a period of 6 months based on each intervention. Quality-adjusted life years (QALYs) are the most commonly used outcome metric in international economic evaluation studies. The selected interventions include (1) TAU, in this case drug treatment with a prescribed antidepressant for an acute depressive episode, plus a 21-week maintenance phase of drug therapy after remission of symptoms, (2) CBT (face-to-face) with a clinical psychologist for an acute depressive episode, plus a 21-week maintenance phase of monthly booster sessions after remission of symptoms, and (3) the myCompass program for an acute depressive episode, plus a 21-week maintenance phase consisting of a booster Internet-delivered program (eg, mobile monitoring of symptoms, behaviors, and lifestyle factors).

The assumptions of the model are as follows. Individuals can move sequentially between mutually exclusive health states: depressive episode, remission, and maintenance [18]. All patients begin in the “episode” state and receive either myCompass, TAU (antidepressant therapy), or face-to-face CBT over the course of a 7-week period. During maintenance therapy, all patients have consultations with a general practitioner (GP) at prescribed intervals to monitor symptoms, side effects, and compliance. Active relapse prevention in primary care is considered effective [25]. Patients not complying or not responding to either myCompass or CBT discontinue treatment or switch to receive TAU for the remaining cycles in line with accepted stepped-care protocols, that is, from a low-intensity to a higher-intensity level of intervention. Patients receiving first line antidepressants may switch to a different type if they are non-responsive to initial treatment or if treatment is discontinued for any reason (ie, non-response, adverse events). Those who switch treatment from CBT, TAU, or myCompass may either enter the remission state or discontinue treatment. Patients may become non-compliant or drop out due to side effects or lack of response from any state.

The model was constructed in TreeAge Pro Version 2013 (TreeAge Software Inc.). To estimate QALYs generated by each cohort (people receiving myCompass, face-to-face CBT, antidepressants), the time spent in each health state was multiplied by a health-state utility weight corresponding to a quality of life adjustment for a given state of health, where one is perfect health and zero is death. Utility data were obtained from a published study in which patient-assigned health state utilities were reported by clinical response [15]. Estimated resource utilization data were then combined with the relevant unit cost information to give the reference cost associated with each treatment. All treatment costs were adjusted for patients not completing treatment.

The time horizon for evaluating the benefits and costs of interventions for depression was 28 weeks to capture the initial phase of treatment (7 weeks of the myCompass program, up to a maximum of 10 sessions of CBT, or antidepressants at recommended initial dosages), followed by treatment maintenance after remission, based on current depression treatment guidelines recommending 6 months of treatment at sufficient level to maintain remission [19]. Limited information from published trials on the longer-term consequences of online intervention use was available beyond this timeframe. Additionally, the length of time to continue antidepressant treatment beyond 6 months after recovery from a depressive episode remains unclear [28]. In Australia, Internet-delivered or e-mental health (e-MH) programs can be accessed directly by the public through specialized or general online portals. Alternatively, e-MH can be recommended as a first step by GPs. Our model takes the latter route into consideration. Model input parameters are listed in Table 1 [8,15,20,29-37] (see Multimedia Appendix 1 for calculation methods indicated).

**Table 1:** Model input parameter values and sources of information.

Parameter		Value	Uncertainty distribution	Sources and assumptions
**Effect size – Initial**				
	Antidepressants	0.462	Beta (121, 140)	[29] Review of clinical trials
	CBT	0.479	Beta (127, 139)	[29] Review of CBT clinical trials
	*MyCompass*	0.449	Beta (202, 247)	[8] Clinical trial data
**Effect size – Maintenance**				
	Antidepressants	0.374	Beta (1075, 1801)	[30] Clinical trial data
	CBT	0.559	Beta (146, 115)	[31] Meta-analysis of CBT clinical trials
	myCompass (follow-up)	0.349	Beta (122, 228)	[8] Clinical trial data
	Return to treatment	0.4		[37]
**Probability of remission after switch**				
	Second line antidepressants	0.306	Beta (440, 999)	[20] Clinical trial data
	After CBT	0.585	Lognormal (-0.713, 0.188)	[32] Meta-analysis results
**Non-adherence**				
	Antidepressants	0.163	Beta (599, 3072)	[20] Clinical trial data
	CBT	0.222	Beta (59,206)	[20] Review of clinical trial data
	myCompass	0.279	Beta (201, 519)	[8] Clinical trial data
**Non-adherence (maintenance)**				
	Antidepressants	0.336	Beta (1436, 2839)	[34] Values from retrospective database analysis
	CBT	0.184	Beta (962, 4268)	[33] Meta-analysis of discontinuation trial data
	myCompass (follow-up)	0.486	Beta (350, 370)	[8] Clinical trial data
	Resource use			
	GP visits/cycle			
	Episode	2.48		[35] Average number of visits per patient; Longitudinal database analysis^b^
	Remission	1.89		[35] Average number of visits per patient; Longitudinal database analysis^b^
	Psychiatrist visits/episode	0.056		[36] Average number of psychiatric consultations for depression; Population survey data^b^
**Utility for depression**				
	Mild	0.78 (0.20)	Beta (15.74, 4.44)	[15]
	Moderate	0.58 (0.31)	Beta (0.88, 0.65)	[15]
	Maintenance	0.88 (0.22)	Beta (1.44, 0.19)	[15]

^a^Weighted average annual costs of combined services.

^b^See Multimedia Appendix 1 for calculation method.

### Costs

With respect to cost inputs, estimates were made of the costs of each arm (myCompass, CBT, and TAU). Estimates of resource use and the unit costs of these resources were obtained from the literature and administrative data and are shown in Table 2. A provider-based perspective, that of the Australian health provider (Medicare and the Pharmaceutical Benefits Scheme), was adopted and thus only direct costs (medication, health service use) were considered. Intervention costs included service provision costs and medication costs (Table 2). This assumes that development and maintenance costs of running e-MH programs are either transferred from current providers to government or are subsidized. Costs for each health state (depressive episode, remission, and maintenance) were estimated by multiplying the number of units of each resource consumed by the estimated unit cost of each resource and then summing the products across different resources. All cost data were for the year 2013/14. No discounting of costs or benefits was necessary since the overall time horizon of the analysis was less than 12 months.

**Table 2:** Resource unit costs.

Model input	Unit cost	Source	Assumptions
GP Mental Health Plan	71.70	MBS items 2700, 2712 (review)^a^	*Mental Health Plan items (initial and review). Statistics are based on relevant MBS items processed from April 2013 to March 2014 [38]*
GP consultation	36.88	MBS item 3, 23^a^	*Cost of a standard GP consultation [38]^b^*
Psychiatric consultation	367.80	MBS items 291, 293^a^	
Psychologist	141.87	MBS items 80000, 80010^a^	Cost of a single session, based on weighted average cost of CBT items [36,38]
Course of CBT	737.72	MBS items 80000, 80010^a^	Based on weighted average cost of CBT MBS items processed from April 2013 to March 2014 multiplied by average number of sessions attended [36,38,39]
myCompass	56.39		Cost of delivery, derived from budgeted delivery costs per user as is currently dispensed (12-month costs)

^a^Weighted average annual costs of combined services (MBS=Medicare Benefits Schedule).

^b^Cost of a follow-up consultation once mental health plan has been implemented.

The costs input for the myCompass arm of the model were derived from the costs associated with delivery of the myCompass program (including enhancements, debugging, server licence, security adjustments, and administration) per user as it is currently dispensed, and service delivery costs. For TAU as a first or second line of therapy, medication costs were varied by averaging the least and most costly medication among the most commonly prescribed antidepressants according to administrative data (Australian Government, 2012) [40]. Services provided by clinical psychologists were assumed to be funded by the public insurance scheme (Medicare) and costed according to the average weighted cost per session multiplied by the median number of sessions attended based on reported findings from an administrative dataset [36,38]. Although myCompass is designed to be a self-help program, for the purposes of this analysis, we took a conservative approach. All three interventions were assumed to require an initial visit to a GP for a mental health plan and referral to either a psychologist or psychiatrist, with a follow-up GP consultation at the conclusion of a course of treatment thus incurring the respective costs for preparation of a mental health plan and standard GP consultation. Those in the CBT arm incur costs related to psychology consultations, that is, for either a course of CBT or single booster sessions during the maintenance phase of treatment. Although mild depression is far more prevalent than moderate or severe depression in the general population, the degree of severity influences the proportion of people presenting to health services for treatment, with those with moderate symptoms presenting to services at approximately twice the rate (68.8% versus 31.2%) [41]. These proportions were factored in to QALY calculations by multiplying these proportions by their respective utility values. Additionally, based on Australian survey data, people with depression present for psychiatric consultations at a rate of between 0.3% and 10.6% on a monthly basis [36]. We used this data to estimate the rate at which depressed patients would be referred for psychiatric services [42]. Resource use is related to the response level for each intervention and the resulting state transition (to remission, maintenance, or relapse to episode). For parity, we assumed an equal likelihood of acceptability to patients for each intervention in the model, although in reality levels of acceptability may differ. To determine costs for each state, we assumed the same mix of providers and respective resource use as reported in longitudinal database and population survey data [35,36,38]. For example, patients experiencing an acute depressive episode require on average between 2-3 GP visits per cycle.

### Model Assumptions

The model relied on a number of assumptions. First, if an initial treatment was ineffective, the patient was switched to TAU, which in this case was antidepressant medication. Second, success rate was independent of previous treatment exposure. This assumption is consistent with current depression treatment guidelines, which have shown that response to one antidepressant does not help predict responses to another class of drug [21]. Therefore, we assume the same efficacy for all antidepressants.

### Analysis of Uncertainty and Sensitivity Analyses

Probabilistic sensitivity analyses (PSA) were conducted to determine the effect of all variable uncertainty simultaneously within the model using data described in Table 1. The probability distributions around the input variables are based on either standard errors or a range of parameter values as published in or calculated from the literature. A simulation of 10,000 runs generated a joint distribution of cost and effect pairs. The effectiveness of treatments was expressed in terms of QALYs. All patient-level data (transition probabilities, costs, utility values) were entered as prior distributions and not as point estimates, enabling random re-sampling and the characterization of parameter uncertainties.

Incremental costs and effects were calculated for the intervention and the comparators, and incremental cost-effectiveness ratios (ICERs) determined to compare costs and effects using the formula, ICER = ΔC/ΔE. This describes the ratio of the change in costs of the intervention compared to each comparator to the change in the effects of the intervention [43]. As ICERs can compare only two groups, Net Monetary Benefit (NMB) was calculated for each group by multiplying the change in QALYs by AUD 50,000 per QALY and then deducting change to costs using the formula, NMB = E*WTP-C (where E represents effectiveness, C represents cost, and WTP is the decision makers’ threshold ICER) [43]. This threshold is somewhat arbitrary; in Australia, the Pharmaceutical Benefits Advisory Committee does not explicitly state a cost-effectiveness threshold value. An approximate value of AUD 64,000 has been suggested [44] but may vary [45-47]. The average NMB across 10,000 simulations was calculated along with 95% credible intervals to represent the uncertainty in the decision. A decision that returns a positive NMB is considered to be cost-effective [48], and the optimal strategy is defined as the strategy with highest expected net benefit.

For the three strategies considered in this study, results of the PSA are presented graphically by cost-effectiveness acceptability curves (CEAC). The CEAC of each strategy was obtained by evaluating the percentage of simulated values where the strategy had the highest NMB, as a function of the willingness to pay (WTP), λ.

Univariate sensitivity analysis was completed for all of the model input parameters in order to investigate the effect of individual assumptions on each intervention on the uncertainty around model outcomes. This enables the identification of which parameters are the key drivers of the model’s results. The list of model parameters and their associated sampling uncertainty included in the 1-way sensitivity analysis are shown in Table 1.

### Scenario Analysis

The cost of providing myCompass in the model is an average based on estimates of support costs and numbers of program users from 2012-2014. Thus costs of delivering an e-MH intervention can vary—volume savings arise as the number of patients treated increases over time. As such, we performed a threshold analysis to determine the maximum cost at which the implementation of myCompass is no longer cost effective.

### Value of Information Analysis

We conducted an evaluation of the Expected Value of Perfect Information (EVPI). The EVPI estimates the difference between the expected value of a decision with perfect information and the expected value of a decision given the current evidence base (at a WTP threshold of AUD 50,000 per QALY) over a period of 1 year. This gives an estimate of the maximum value of further research [49]. Decision makers faced with the findings of research have to appraise the available evidence base and decide if a new technology should be adopted into clinical practice on the basis of existing information due to the opportunity costs of making the wrong decision when the evidence base is not sufficiently certain [49]. We then estimated the Expected Value of Perfect Partial Information (EVPPI) to assess the value of uncertainty around model parameters. To estimate the EVPPI with accuracy [50], we ran an optimal number of iterations for each analysis.

## Results

### Sensitivity Analysis

**Figure 2:**
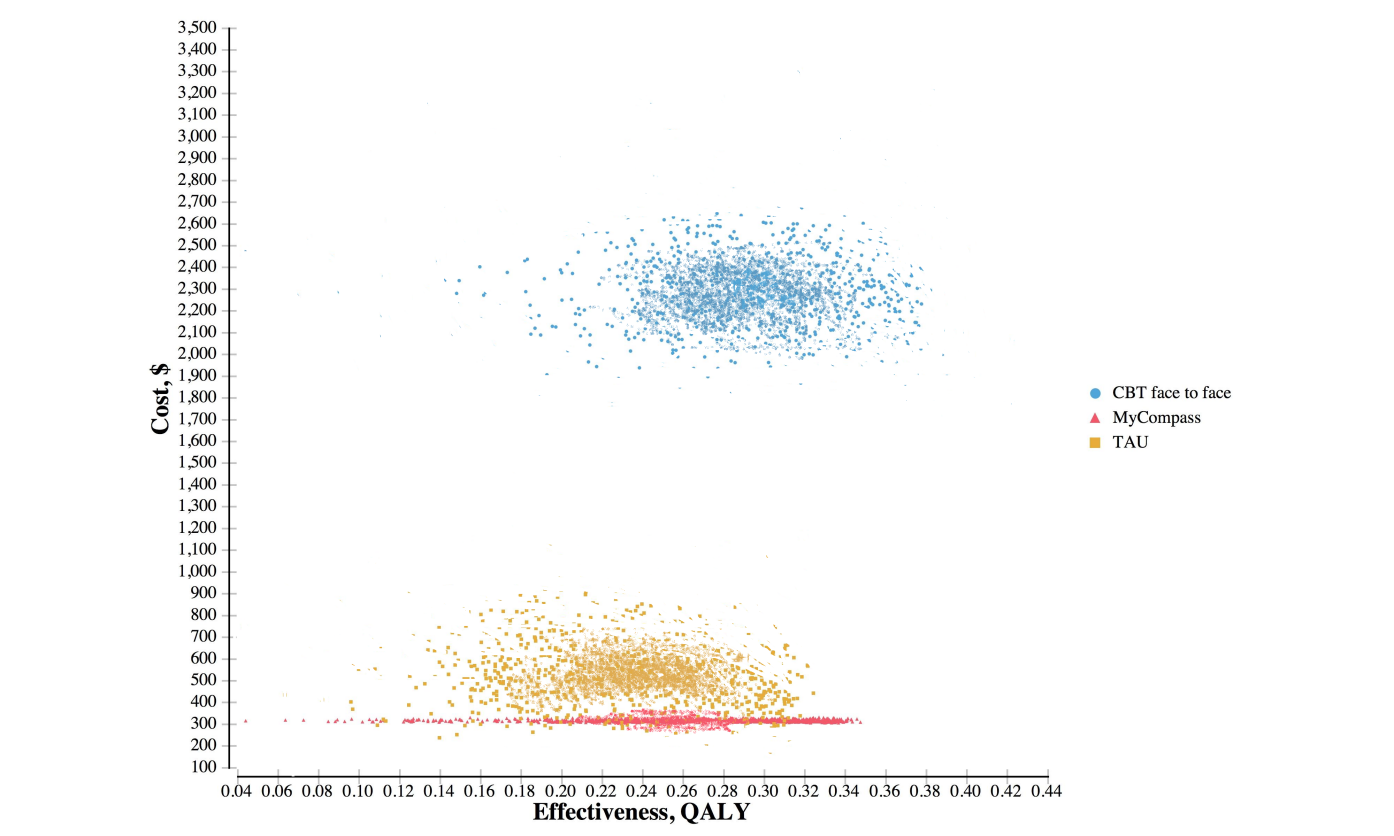
Sensitivity analysis - cost-effectiveness plane.

The average NMB across 10,000 simulations was calculated along with 95% credible intervals to represent the uncertainty in the decision. Table 3 provides the estimates of the costs and QALYs generated by the baseline configuration, identifying myCompass as the strategy with the highest net marginal benefit. The resulting cost-effectiveness plane can be viewed in Figure 2. The mean incremental NMB per individual (ie, the average NMB for myCompass minus average NMB for comparator) for the myCompass group compared to the TAU group was AUD 1165.88 for the TAU model and AUD 522.58 for the CBT model. The incremental cost relative to myCompass was AUD 190 per individual for the model using TAU as the first line of treatment and AUD 1995 per individual for the CBT model (Table 4).

**Table 3:** Results of PSA, showing differences in costs, health benefits gained, and net monetary benefits.

Model statistic	TAU Mean (95% credible interval)	CBT Mean (95% credible interval)	myCompass Mean (95% credible interval)
Cost	524.91 (457.05-619.77)	2330.51 (2201.10-2408.40)	334.96 (332.01-338.75)
QALYs	0.24 (0.15-0.32)	0.29 (0.16-0.37)	0.26 (0.15-0.34)
Av. CER	2187.13	8036.24	1288.33
NMB	11308.61 (6881.53-15514.74)	11951.91 (5158.75-16254.58)	12474.49 (6521.75-16599.58)

**Table 4:** Incremental results, myCompass versus comparators.

	TAU	CBT
Incremental costs	158	1996
Incremental QALYs	-0.02	0.03
ICER	-8425.37	2966.37
Incremental NMB	-1165.88	-522.58

**Figure 3:**
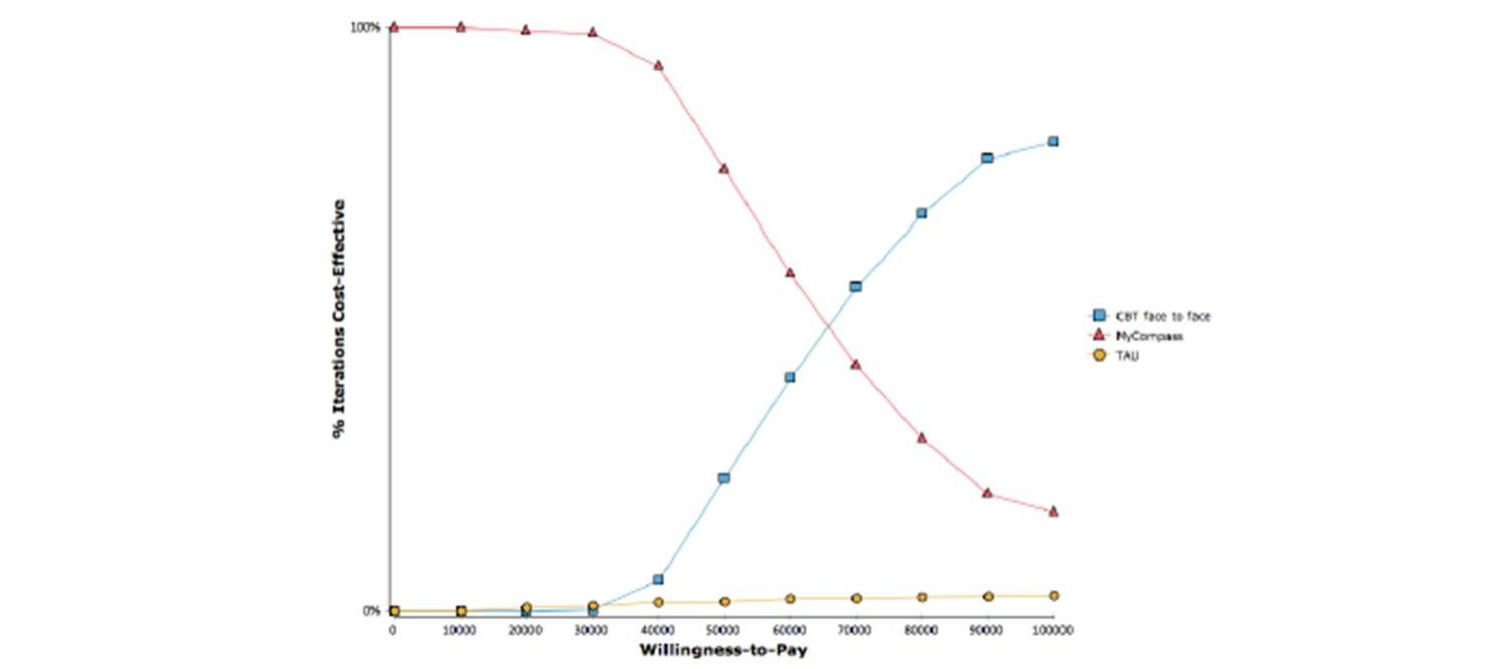
Cost-effectiveness acceptability curve: myCompass versus CBT and TAU.

Figure 3 presents the PSA results in the form of a CEAC, showing the proportion of the costs and effects pairs that are cost-effective for a range of values. This gives an estimate of the proportion of the simulated distribution of cost and effect pairs that lie below a given threshold of AUD 50,000 (the maximum value a decision maker is prepared to pay for a unit of effect), that is, the proportion that generates positive net (monetary) benefits. Figure 3 shows the probability that myCompass is cost-effective compared with CBT and TAU at a WTP threshold of AUD 50,000 (75.5%). The probability that CBT is the most efficient strategy increased as the threshold value increased, whereas myCompass is the favored strategy for 96.7% of threshold values when compared to TAU. At a WTP of 0, there is a 100% likelihood that myCompass is the most cost-effective strategy. At the selected WTP threshold, a proportion of iterations were less costly but also less effective than comparators (22.9% versus CBT, 1.6% versus TAU). Thus the CEAC moves from 100% toward a minimum of 17%—a decreasing function of the WTP. The CEAC corresponding to TAU forms a horizontal line at close to 0%, representing the dominated case. Conversely, in the case of CBT, the CEAC reaches a maximum value of 80.3% and becomes the most efficient option at a WTP of AUD 65,000.

### Uncertainty Analysis

The variables with the greatest impact on the NMB are shown in Table 5. The sensitivity analysis with most deviation from the base-case analysis was the non-adherence rate of myCompass*,* followed by the cost of delivering the intervention. In univariate analysis, myCompass was no longer cost-effective at AUD 50,000/QALY when the cost of delivering CBT dropped below AUD 309.50 per patient. The average cost of providing myCompass had to rise approximately 5-fold before significant changes in cost-effectiveness comparisons were noted. Table 5 shows the lowest and highest expected value for the model based on the optimal strategy at each point.

The distribution of cost-effectiveness results is summarized by the cost-effectiveness acceptability curve (Figure 3).

**Table 5:** Results of uncertainty analysis.

	Range in variation of parameter	Low expected value (NMB)	High expected value (NMB)	Threshold at which myCompass is no longer the most efficient strategy
Probability of non-adherence myCompass	0.0-0.35^a^	12,365.63	13,059.98	—
Cost of delivering CBT (AUD)	0.0-619^a^	12,421.34	13,034.60	309.50^b^
Cost of delivering myCompass	0.0-564	12,005.16	12,682.00	281.95^b^

^a^Based on 95% confidence intervals for non-adherence and remission rates and 0 to +20% of base-case value for cost of delivery of CBT.

^b^myCompass less efficient below this value.

###  Expected Value of Information

Results of the EVPPI analyses suggested that the rate of non-adherence for myCompass was the single parameter for which value of further information might be obtained. Other parameters had an EVPPI of 0. The reasonably high level of confidence in the cost-effectiveness results at the WTP threshold reflect this finding. We estimated the expected per-patient value of perfect information to be AUD 79.37. We extrapolated this to a population level by estimating the number of patients that would be likely to receive the intervention over a 1-year period. The current estimates of the prevalence of mental illness in Australia is 20.1 % across all age groups (4.4 million) [51]; of those, 20.6% have a mood disorder [41]. Approximately 2 million people with a current mental disorder present to health services [51]. Treatment rates for people seen by health services by level of severity is around 25% for mild and 59% for moderate [41]. Hence, based on these data, assuming that both the prevalent and incident population are treated, based on an acceptability rate scenario of 2%, we estimate the eligibility for the intervention to be ((25%+59%)*(20.6%)*4.4 million) at total coverage. Assuming a scenario with a low acceptability rate of 2% [52] (of those with mild-to-moderate levels of disorder), at a population level, the EVPI for the intervention is approximately AUD 1,208,608. This represents a maximum estimate of the value of further research if perfect information were achievable for all model inputs.

## Discussion

### Main Findings

This study shows that implementation of the myCompass program is potentially cost-effective in the Australian setting, reducing treatment costs to providers without evidence of diminished treatment efficacy. Internet-based stepped care, and the impact of such care for depressive symptoms, has not been extensively studied and economic evaluations of this type of model are scarce. Our findings show that a stepped-care approach, with Internet-delivered, unguided self-help as the first step, may be a useful and cost-effective way to improve access to treatment for depression.

The cost of delivering CBT remains relatively high. Because of the cost benefits, research into the factors that influence use of Internet-delivered programs is urgently needed. Meta-analyses and reviews show that outcomes and adherence rates vary widely depending on the method of program delivery, that is, whether the intervention is clinician-assisted, has administrative support, or is unsupported [10,53-58]. However, patient variables are likely to be influential also. For example, patients with higher levels of motivation and less severe depression may be more suited to Internet-delivered treatments because they have the requisite cognitive skills, learning style, and self-regulation needed to complete a treatment course. Non-adherence to the myCompass program was an issue in the trial and hence the cost-effectiveness analysis. Although non-adherence may have implications for upscaling this intervention, qualitative evidence has shown that in many cases, patients withdraw for personal reasons such as improvement in symptoms (known as “e-attainers”) [59,60], not because of problems with the technology or the social environment [61]. Including programs such as myCompass as part of an integrated model allows clinicians to monitor interim outcomes and inform decisions on patient treatment pathways, in case some patients are negatively affected by the limitations in effectiveness of low-intensity treatments. In this way, public health programs become more comprehensive, strategically leveraging existing knowledge, infrastructure, and resources to improve health outcomes.

Our analysis was, however, exploratory, and some caution is warranted in interpreting our findings as the model had several limiting assumptions. In this study, a series of univariate sensitivity analyses explored the impact of varying all resource costs, probabilities, and utilities on the incremental cost-effectiveness of myCompass compared to TAU and CBT. The cost-effectiveness of myCompass in this model was largely dependent on the probability of discontinuation of myCompass and, to a lesser degree, the delivery cost of the program, as opposed to findings from other literature where delivery costs of CBT were major cost drivers [14,62]. The EVPPI analyses suggested that at a WTP threshold of AUD 50,000 per QALY, there is reasonable certainty that myCompass will be cost-effective, regardless of which parameter value is taken within the bounds of the modeled distribution. Although the cost of delivery of myCompass in our model was considerably lower than other similar programs, such as that offered by the US National Stress Clinic [63], threshold analysis showed that even a substantial rise in the implementation cost of myCompass had little impact on cost-effectiveness. Our EVPI analysis suggested that the non-adherence rate for myCompass is the factor that would benefit from future research. High rates of discontinuation from Internet therapy programs have been noted in several studies [13,64-67], with lack of motivation in depression remediation being a possible contributor. On the other hand, there is evidence that participants who show early symptom improvements may discontinue program use because they feel it is no longer needed [40]. The estimated remission rate for myCompass was characterized using a Beta distribution. The parameters of this distribution were based on completer analysis and thus may be subject to attrition bias as it is possible that some of those lost to follow-up may have experienced a relapse in their depression (or a remission). Consequently, while our PSA characterized the uncertainty observed in the myCompass trial, the characterization of some parameters and the resultant dispersion of the distributions may have been affected by incomplete data.

### Implications

In line with previous studies, our findings showed modest but comparable effectiveness for myCompass versus CBT, and favorable QALY outcomes versus TAU. Previous studies have found similarly moderate results in incremental effectiveness to those reported here [11,13,14,16] but also found that the majority of costs were attributable to productivity and societal costs, with higher ICERs. The association between depression, disability, and lost productivity due to illness is well established [68]; days missed from work due to mental illness decline significantly when remission of depression is attained. Given the high incidence of depression among people of working age, precluding productivity costs could be considered a limitation of the study as interventions that have a strong effect on the productivity of the working population may produce productivity costs that reflect a large part of total costs. Thus, the inclusion of productivity costs may cause incremental costs to change from positive to negative, or vice versa, depending on study design factors such as time horizon, methodology used to measure lost productivity, and type of treatment. Only one study [13] analyzed the variations in societal cost calculations but found this produced similar CEACs. When considered from the perspective of the provider (as taken here), lower health services costs among those recovering from depression are commonly observed [69]. Considered from the societal perspective (that is, considering indirect costs such as lost productivity), a trend toward lower health services costs and reductions in lost work due to illness were noted.

This being the case, it is reasonable to assume that with reduced costs of care, lower implementation costs, greater efficiencies due to minimal therapist contact, and increased reach and access, implementation of e-MH interventions into routine care may have collateral benefits such as reductions in direct costs to stakeholders and greater treatment parity. Further cost savings are likely to arise due to the extended reach and fidelity conferred by Internet-based interventions, and the reasonable probability (based on current uptake of available programs [12], plus unmet need for services) that such interventions will find a sizeable target population. As Internet-based interventions have shown to be generally acceptable to patients [70], greater adherence might also arise with increased familiarity and changing perceptions and attitudes (therapeutic alliance, confidentiality) among health professionals and the general public. Potential indirect cost savings may include increased health knowledge in users and ability for self-care. These additional outcomes need to be tested empirically. However, the data presented here are sufficient to indicate that e-MH programs as treatments for depression could have a clinically meaningful and cost-advantageous impact at a larger scale. Thus, the cost-effectiveness of e-MH programs could fall well below the current WTP threshold for implementation than demonstrated in this analysis. Analysis of a United Kingdom stepped-care program similar to that proposed here concluded that it was cost-effective within the National Institute of Clinical Excellence threshold range of £30,000 per QALY; the program continues to form an important part of mental health care provision [71].

### Strengths and Limitations

Our findings are based on data derived from a large community-based RCT, in which participants were recruited nationally via a range of media, including websites, social media channels, print media, and corporate and government organizations. That the data have high external validity with respect to extrapolation of effectiveness at the population level is a strength of this research. The model chosen to examine the relative cost-effectiveness of these interventions was one based on stepped care. We acknowledge that a stepped-care approach may not be optimal for all individuals, particularly those in crisis, or those with comorbid, or complex needs. Nevertheless, the stepped-care model represents an accepted approach for people with mild-to-moderate common mental health disorders. It would be possible to do more sophisticated modeling of different scenarios into the future.

A number of important sources of costs associated with depression were not included in our model. For example, the model did not include the potential utility decrements associated with adverse effects of antidepressants, inclusion of which may have reinforced the cost-effectiveness of both psychologically based therapies. A narrow provider perspective was adopted and did not include drug dispensing costs or downstream cost offsets. Indirect costs of depression such as productivity losses, presenteeism, and intangible costs to patients (eg, unrestricted access to treatment) were also not considered, although these values are important from a societal perspective and may have led to an underestimation. The short 6-month timeframe of the model was limited by data available from the existing clinical trial of myCompass. With regards to structural uncertainty, our model may represent a simplification of the progression of disease and clinical presentation in primary care. As depression can be a chronic condition, four cycles may not depict the full scope of cost-effectiveness comparisons between the three interventions. For example, our model did not allow for treatment enhancement such as combining medication and CBT, which would otherwise be part of a stepped-care strategy. However, without additional data, the use of a longer timeframe would require assumptions on the outcomes of the intervention and introduce further uncertainty. As myCompass is designed to be used without clinician consultation, it is possible that costs were overestimated. However, a recent meta-analysis found larger effect sizes for psychological therapies in patients referred by their GP as opposed to those recruited through screening [72]; it is possible that users are more adherent when interventions are integrated into primary care, impacting on costs. We did not include costs associated with introducing stepped care such as training of primary care providers and the establishment of referral networks between mental health care providers, despite research showing that factors such as unfamiliarity with eHealth instruments and websites, appropriateness of interventions, and uncertainty around multidisciplinary collaboration provide major barriers to uptake in international settings [73]. We did not factor in lag times to treatment that are likely to occur in clinical practice.

### Conclusion

Health services internationally are currently challenged in disseminating care for depression. Internet-delivered interventions can provide access to treatment to those who would otherwise either not receive it or be placed on waiting lists or ineffective medications.

Widespread dissemination of e-MH interventions can potentially reduce demands on primary and tertiary services and reduce unmet need. This analysis adopted a decision tree model to estimate how the adoption of e-MH would impact the costs and outcomes of allocation of treatments to patients in routine care in the Australian health system. We found that implementation of an e-MH program (myCompass) was cost-effective compared to usual care and face-to-face CBT. Further research is needed to determine other related factors including population effectiveness and how implementation costs would be distributed across various stakeholders.

## Appendix

Multimedia Appendix 1 Calculation of resource use.

## Abbreviations

CBT: cognitive behavior therapy
CEAC: cost-effectiveness acceptability curve
e-MH: e-mental health
EVPI: expected value of perfect information
EVPPI: expected value of perfect partial information
GP: general practitioner
ICER: incremental cost-effectiveness ratio
MBS: Medicare Benefits Schedule
NMB: net monetary benefit
RCT: randomized controlled trial
TAU: treatment-as-usual
WTP: willingness-to-pay
