# Supplementary material for: e-CBT (myCompass), Antidepressant Medication, and Face-to-Face Psychological Treatment for Depression in Australia: A Cost-Effectiveness Comparison
Source: J Med Internet Res. 2015 Nov 11;17(11):e255. doi: 10.2196/jmir.4207 (PMC4704984; doi:10.2196/jmir.4207)
Supplement: Multimedia Appendix 1 [file jmir_v17i11e255_app1.pdf]

Appendix 1

Calculations for resource use

Our calculations for this input were based upon a longitudinal analysis of a large United Kingdom primary care general practice database (Byford 2011, ref.33). This database measured health service use for patients treated for depression by remission status. The analysis found that those with remitted depression had fewer GP (13 versus 17) and psychiatry consults (40% versus 47%) than those with non-remitted depression over a 12 month period. We estimated a rate of approximately 1.42 GP visits for a depressive episode and 1.1 visits for a remitted episode over 7 weeks:

17/12*7/4=2.48 (probability=0.881)

13/12*7/4=1.89 (probability=0.803)

For psychiatric visits (Goldney 2004, ref.34):

Over a one month period -

10.6% of 205 people with major depressive disorders saw a psychiatrist =20

3.2% of 319 people with ‘other’ depression’ saw a psychiatrist =9

0.3% of 389 people with subsyndromal depression saw a psychiatrist=1

Total visits approximately 30/913=3.2%

Converting this to a probability:

Probability of visiting a psychiatrist over a 7 week period for the treatment of depression

=(1-(EXP(-1*(0.032*7/4)))) = 0.054

Converting the probability into a rate:

-1/t*LN(1-p)=0.056
